# The positive impacts of early-life education on cognition, leisure activity, and brain structure in healthy aging

**DOI:** 10.18632/aging.102088

**Published:** 2019-07-17

**Authors:** Yaojing Chen, Chenlong Lv, Xin Li, Junying Zhang, Kewei Chen, Zhongwan Liu, He Li, Jialing Fan, Ting Qin, Liang Luo, Zhanjun Zhang

**Affiliations:** 1 State Key Laboratory of Cognitive Neuroscience and Learning, Beijing Normal University, Beijing, P. R. China; 2 BABRI Centre, Beijing Normal University, Beijing, P. R. China; 3 Teaching and Research Section, Graduate School, Academy of Military Sciences, Beijing, P. R. China; 4 Institute of Basic Research in Clinical Medicine, China Academy of Chinese Medical Sciences, Beijing, P. R. China; 5 Banner Alzheimer's Institute, Phoenix, AZ 85006, USA; 6 Department of Psychology, The University of Hong Kong, Hong Kong, China

**Keywords:** education, aging, cognition, brain reserve

## Abstract

Education in people’s early lives are positively related to their cognitive function, but its modulating effects on detailed cognition domains, its interaction with leisure activities and the associated brain changes have yet to be investigated. This report used data from 659 cognitively normal community dwelling elderly who completed neuropsychological tests, leisure activities measurement, and 78 of them underwent structural and diffusion MRI scans. We found that: (i) the highly educated elderly had a better cognitive functioning in multi-domains, higher frequencies of participation in knowledge-related leisure activities, and slower age-related reductions of executive function; (ii) the intellectual and social types of leisure activities mediated the association between education and multiple cognitive domains, including memory, language, attention and executive function; (iii) there was a significant age by education interaction on the gray matter volume of the anterior brain regions and white matter integrity; and (iv) the interaction between age and education affected cognition indirectly through white matter integrity analyzed using structural equation model. Overall, our results revealed that high education in early life served as a protective factor in aging that may help to postpone cognitive and brain reserve decline in cognitively normal aging.

## INTRODUCTION

As we age, the brain and cognitive functions inevitably decline. Various intrinsic and extrinsic factors contribute to the delay or acceleration of this process [1]. Researchers have proposed the cognitive 'reserve' (CR) hypothesis, which suggests that people with higher reserve are more capable of tolerating aging process and related disease, cognitive impairment and injury in brain and cognition [2, 3]. Reserve typically refers to cognitive stimulating experience through life course. Therefore, the educational and occupational attainment were widely used as cognitive reserve proxy [4].

Education is one of the best-established non-medical protective factors for recognition aging [5]. Epidemiologic studies showed highly educated individuals had reduced risk of dementia [6] and cognitive decline [7], compared to their poorly educated counterparts. The CR theory deems that the mental trainings in people’s early lives, mainly in the form of education, offer an additional capacity in cognition to compensate for the decline in the late life [2, 3]. In other words, people with more mental stimulating experience are able to maintain better cognitive function when brain pathology is taking place. Roe and his colleague found that a group of high-educational older adults with neuropathological AD showed no dementia in their lives, in contrast to the low-educational group [8]. These findings have brought a new perspective for understanding the pathogenesis of cognitive aging and dementia, thus providing a non-medical means on prevention [9].

However, the underlying mechanism of the protective effects of education on cognition aging remains obscure. One possible pathway from the perspective of environmental factor, is that education protects cognition through life activity. As the Use It or Lose It hypothesis being raised up, many studies revealed that the mental stimulation (such as leisure activity) in adulthood protects cognitive function in older age [10]. A longitudinal study found that the activity participation throughout adulthood predicted better cognitive ability in older adults, among which leisure activity engagement in middle age positively related to better cognitive function [11]. This is consistent with CR theory that abundant leisure activity in late life makes an individual more resilient to brain aging [12]. Besides early life experience gained from educational attainment, more and more studies also treated life activity in adulthood as one of the effective cognitive reserve proxies [13]. Meanwhile, education is also an indicator of lifestyle [14]. Education in early life may cultivate the knowledge, skills, and ability necessary for continued participation in activities well into later adulthood. Thus, it can be seen that life activity has a very close relation to education, and it may mediate education’s effect. However, this has not been well studied. Although some studies reported association between lower educational attainment and higher dementia, these alleged effects of education may not be specific for dementia but represent an alternative pathway from negative lifestyle to increased incidence of age-related cognitive impairments [14–16].

Another prospective of the topic is in respect of neural basis under education’s protective effect. As the Brain Reserve theory pointed out, sufficient neural substrates (e.g., more brain volume) are capable to bear more brain deficit before it reaches a clinical threshold [17]. Some studies also proved that educational attainment associated brain reserve. Longer education duration is reportedly associated with ameliorations in several brain MRI indexes in older age, such as greater cerebral [18] or gray matter volume [19], and ostensibly more favorable white matter macrostructure and microstructure [20–22]. Additionally, our previous study showed that in healthy elderly, education level was positively correlated with complex cognitive functions, and correlated with regional functional connectivity within resting-state networks relating to complex cognition [23]. However, the results are not consistent and some conflicting ones have shown little support for the brain reserve hypothesis [24].

The conflicting situation may be mainly due to analyses of isolated/regional MRI biomarkers and the absence of an important potential confounder, age, for advanced age is the strongest known risk factor for brain atrophy [25]. However, up to date, only one study highlights the two points, which found that education attenuated the association between aging and brain structure in a large-sized cognitively normal cohort, regardless of vascular risk factors [26]. And issues related to the effects of education on the gray matter (GM) volume and the integrity of white matter (WM) in aging, globally or regionally, have not been well investigated.

The current study examined how the education attainment affects age-related changes in specific cognitive domains and social activity engagements, the interaction between the two, and their interactions with brain gray/white matter structures, based on a sample of 659 cognitively normal community dwelling elderly people in Beijing, China. A comprehensive battery of neuropsychological tests and a personal leisure activity questionnaire were used to assess cognition and social activity engagements. Moreover, we explored the effects of education on age-related reductions in regional GM volume and WM integrity, as well as the moderating effect of education on the relationship between brain structural atrophy and cognition using volumetric MRI and diffusion tensor imaging (DTI) data from a subset of these 659 individuals. The effect of the APOE ε4 genotype was controlled in all analyses in the present study since it affects brain structure and functions in cognitively normal elderly subjects as a major genetic risk factor for sporadic AD [27] and its negative effects appear to be diminished by the education levels [28, 29]. Overall, the current study aims to examine the following hypotheses: 1) highly educated individuals exhibit more engagement in leisure activity and have less age-related cognitive declines compared to those with lower education; 2) the age effects on brain GM/WM changes are mitigated in individuals with higher education levels; and 3) education acts as a moderator between brain structural changes and cognitive function.

## RESULTS

### The effects of age×education on cognitive performance

A total of 659 participants were enrolled in the present study from Beijing Ageing Brain Rejuvenation Initiative (BABRI) database (Supplementary Figure 1). The demographic and clinical characteristics of the participants in the large samples are presented in Table 1. No differences in age or APOE ε4 genotype were found between the high education and low education groups. A higher proportion of males had more education than females. Additionally, participants with a high level of education had significantly better cognitive performances compared to those with low education attainment (Supplementary Figure 2). Among the cognitive scores, only TMT-B and SCWT-A exhibited a significant education×age interaction (Table 1), suggesting a more age-related reduction in the executive function of elderly with less education.

**Table 1:** Demographic and neuropsychological tests of all participants.

	**Low Edu**	**High Edu**	**p(Edu)**	p(Edu*Age)
Num	439	220	—	—
Age	63.59(7.22)	67.25(6.87)	<0.001	—
Gender(M/F)	151/288	92/128	0.027	—
Education(year)	9.49(1.96)	15.26(1.47)	<0.001	—
APOE ε4 (+/-)	67/372	35/185	0.820	—
MMSE	27.97(1.59)	28.37(1.44)	<0.001	0.658
AVLT-delay recall	6.02(2.44)	6.44(2.27)	<0.001	0.306
AVLT-delay total	31.47(8.72)	33.00(8.33)	<0.001	0.451
ROCF-Copy	33.17(3.55)	34.30(1.94)	<0.001	0.242
ROCF-delay recall	13.71(6.31)	14.85(5.51)	<0.001	0.159
CDT	24.49(3.62)	26.04(2.96)	<0.001	0.977
CVFT	45.22(8.34)	48.39(7.78)	<0.001	0.702
BNT	23.10(3.64)	24.57(3.01)	<0.001	0.332
SDMT	34.71(11.01)	39.34(10.97)	<0.001	0.665
Digit span	7.33(1.31)	7.86(1.19)	<0.001	0.217
Backward digit span	4.21(1.24)	4.73(1.34)	<0.001	0.881
TMT-A time (s)	57.29(18.99)	51.52(15.49)	<0.001	0.205
TMT-B time (s)	175.91(70.74)	152.99(48.23)	<0.001	<0.001
SCWT-A Time (s)	27.77(6.82)	25.60(5.47)	<0.001	0.012
SCWT-B Time (s)	37.49(8.58)	37.49(23.65)	0.468	0.409
SCWT-C Time (s)	74.54(18.24)	73.35(23.93)	0.002	0.121
Intellectual activity	60.53(21.97)	70.93(22.84)	<0.001	0.810
Physical activity	52.0(17.80)	56.42(18.96)	0.004	0.278
Social activity	32.17(15.06)	35.95(14.45)	<0.001	0.806

Values are mean±standard deviation or Nos. of participants.

MMSE=Mini-Mental Status Examination; AVLT=Auditory Verbal Learning Test; ROCF=Rey-Osterrieth Complex Figure test; CDT=Clock-Drawing Test; CVFT=Category Verbal Fluency Test; BNT=Boston Naming Test; SDMT=Symbol Digit Modalities Test; SCWT=Stroop Color and Word Test; TMT=Trail Making Test.

### The influence of education levels on leisure activities

Habitual leisure activity levels were grouped according to intellectual, physical and social factors. Scores of intellectual, physical and social types of leisure activities were significantly higher for participants with a high level of education, but no significant education×age interaction was found (Table 1).

Group differences in leisure activities between the elderly with higher education levels and those with lower education levels were compared using a scale that represents a wide range of elderly people’s daily life in China (Supplementary Table 1). The highly educated elderly prefer to participate in knowledge-dependent activities (including reading, writing, attending a senior university, using a computer, doing crossword puzzles, magic cube, or solitaire, and doing Chinese traditional martial arts) and the less educated elderly participated in knowledge independent activities (including playing chess, poker, or mahjong, manufacturing by hand, and owning pets).

### Mediation analysis

**Figure 1:**
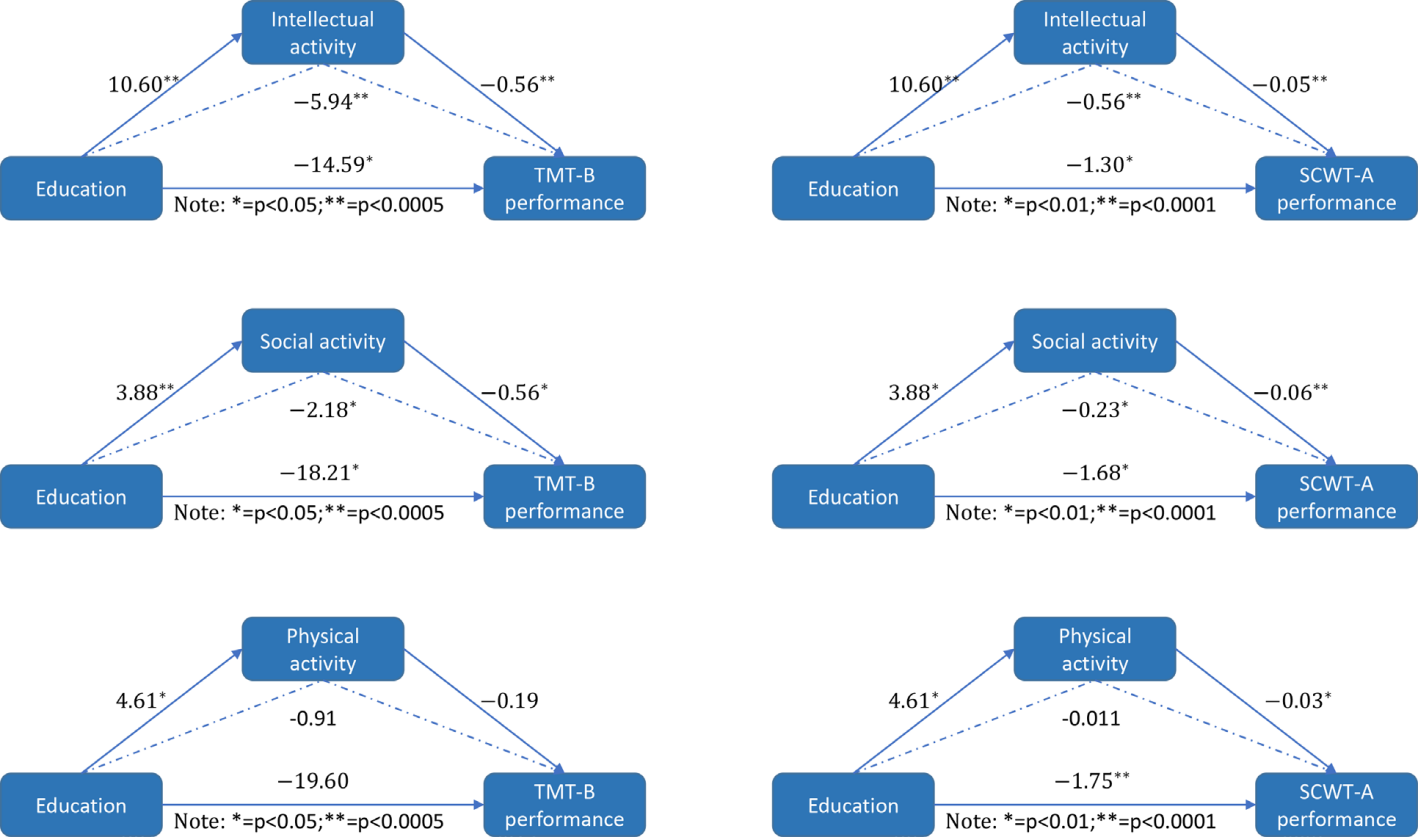
**The mediation model.** The mediation model illustrating the direct effect of education on intellectual/social/ physical activity, the direct effect of education on TMT-B and SCWT-A performance, the direct effect of intellectual/social/ physical activity on TMT-B and SCWT-A performance and the mediating effect of intellectual/social/ physical activity on the association between educational levels and TMT-B/SCWT-A performance, as indicated by the path coefficients and the p values.

In the mediation analysis, the independent factor was the education level and the dependent variables were cognitive measures that showed significant education×age interactions, such as the TMT-B and SCWT-A. The proposed mediators were the intellectual, physical and social activity scores. The mediation analysis indicated that intellectual activity mediates the effect of education on the TMT-B (Z=-3.40, p<0.0005) and SCWT-A (Z=-3.54, p<0.0005) performance, and social activity mediates the effect of education on the TMT-B (Z=-2.00, p<0.05) significantly and SCWT-A (Z=-2.17, p<0.05) performance (Figure 1). Furthermore, we added all the cognitive tests as outcome variable, and we did find some interesting results. The results show that education affects cognitive performance in almost all fields through mental activities, while education can affect attention function, executive function and memory function through social activities. However, we did not find a significant mediating effect of physical activity (Supplementary Figure 3).

### The age×education effects on gray matter volume

**Figure 2:**
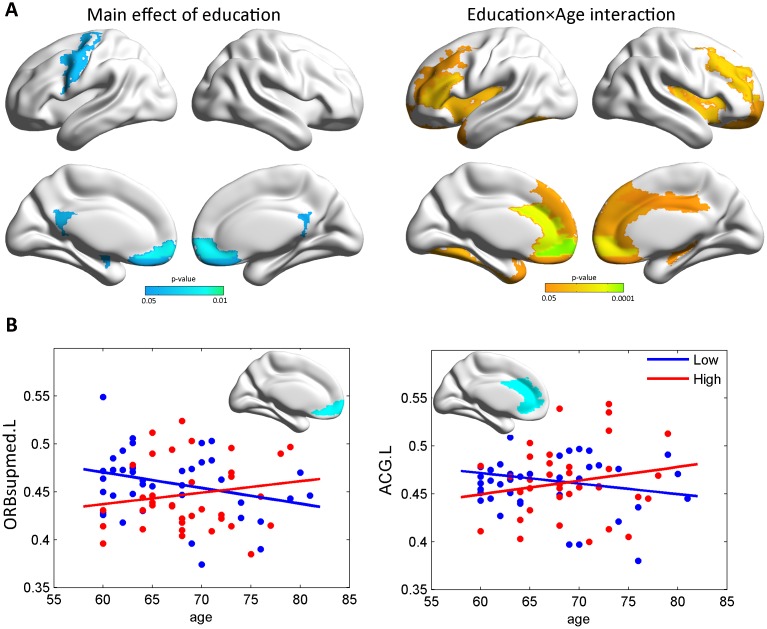
**The age×education effects on gray matter volume.** (**A**) Maps of the significant age×education interaction on gray matter volume. A significant main effect of education on gray matter was also found (p<0.05, uncorrected). (**B**) Scatterplots illustrating the age×education interaction for ORBsupmed.L and ACG.L (q<0.05, FDR-corrected). Specifically, the plots were drawn to illustrate how the GM volume changes within the actual range of age. ORBsupmed.L=left medial orbital superior frontal gyrus; ACG.L=left anterior cingulate and paracingulate gyrus.

The demographic and clinical characteristics of the small samples with available MRI data are presented in Supplementary Table 2. A main effect of educational status was found on the left precentral gyrus, the bilateral medial orbital superior frontal gyrus (ORBsupmed), the bilateral rectus, the bilateral posterior cingulate cortex, and the left amygdala (p<0.05, uncorrected, Figure 2A). After gender and APOE ϵ4 status were regressed out, significant age×education interaction effects were found in several frontal, temporal and occipital regions (p<0.05, uncorrected, Figure 2A), including the ORBsupmed.L, the left anterior cingulate, and the paracingulate gyrus (ACG.L), which persisted after false-discovery rate (FDR) corrections (Figure 2B). There was a trend toward higher age-related GM atrophy among the less educated elderly compared to the more educated elderly in these regions.

### The age×education effects on white matter integrity

**Figure 3:**
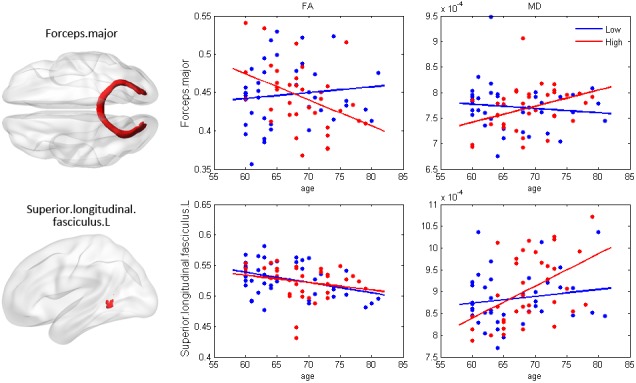
**The effect of age×education on white matter integrity.** The fibers in red exhibited significant age×education interaction effects (p< 0.05), including the FA and MD of the forceps major, and the MD of the SLF.temporal part. The plots were drawn to illustrate how the white matter changes within the actual range of age. FA=fractional anisotropy; MD=mean diffusivity; SLF=superior longitudinal fasciculus.

Similarly, we investigated the interaction effects on the fibers. The main effect of education was significant only on the left cingulum (hippocampus part) mean diffusivity MD value. The MD of the forceps major and left superior longitudinal fasciculus (SLF, temporal part) were affected by the age×education interaction, while the FA was affected only in the forceps major, as shown in the scatter diagram (p<0.05, uncorrected, Figure 3). Our post-hoc analysis found that the years of education was negatively correlated with the MD values of the forceps major and the SLF.temporal part (Supplementary Figure 4).

### Structural equation modeling

The results above demonstrate that the interaction of education and age has effects on both cognitive performance and brain structure. We further tested whether the brain-cognition relationship was also influenced by the age×education interaction. We analyzed the data using structural equation modeling to discuss the relationship between education, WM, GM and cognition, as shown in the following figure. Please notice that the only the MD value of the SLF. Temporal part was included in the model as the WM latent variable. The reason why we only considered SLF.temporal part (MD value) instead of the three white matter indicators was because the poor relationship among the three indicators made it unreasonable to extract a latent variable, so it wouldn’t make sense if the three indicators were considered together in one model. Instead, we modeled on each one separately, and only modeling on SLF.temporal part (MD value) revealed a meaningful model fit (RMSEA=0.0468, NFI=0.934, CFI=0.989, GFI=0.963, AGFI=0.868).

**Figure 4:**
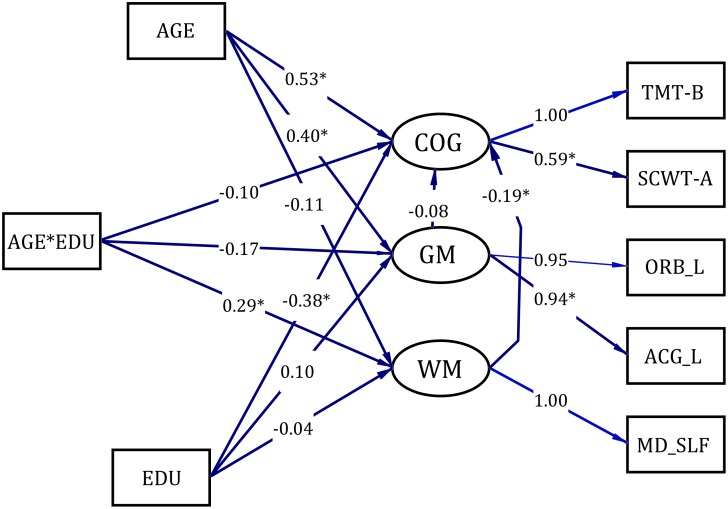
**The structural equation modeling of the relation among white matter, grey matter and cognition**. COG= Cognitive Tests; GM=gray matter; WM=white matter; ORB_L= left medial orbital superior frontal gyrus; ACG_L=left anterior cingulate and paracingulate gyrus; MD_SLF= the mean diffusivity of the SLF.temporal part; SCWT-A=Stroop Color and Word A Test; TMT-B=Trail Making B Test.

As shown in Figure 4, the first path of each measurement model was latent variable scaling, and the values on each path were marked with the estimated values of standardized parameters of the path. Results indicated that education had a significant main effect on cognition only, but not on GM or WM. The interaction between age and education, on the other hand, had a significant effect on WM, but not on cognition or GM. However, the significant effect from WM to cognition was detected, indicating the interaction between age and education could affect cognition indirectly through SLF.temporal part.

## DISCUSSION

In the present study, we aimed to explore the effects of education on cognitive aging and brain aging in cognitively normal elderly. The main findings include: (1) cognitively normal elderly with high educational attainment have a high level of wide cognitive functioning and a significantly decreased age-related reduction in executive function; (2) the intellectual and social types of leisure activities mediate the association between education and multiple cognitive domains, including memory, language, attention and executive function; (3) there are differences in age-related GM atrophy between the high and the low education groups in the anterior regions (ORBsupmed.L and ACG.L) and age-related WM damage in the forceps major and SLF.temporal regions; and (4) the regional WM integrity mediates the interaction effects of education and age on cognition.

The concept of CR suggests that more education may help delay cognitive declines in the healthy elderly, and may also enable individuals with AD to be more resilient to brain damage by allowing them to use cognitive processing or compensatory approaches [30, 31]. In the current study, individuals with higher educational attainment performed better in almost all of the cognitive domains including visuo-spatial ability, executive function, and language. Our findings confirmed those obtained in other studies exploring the role of education in healthy participants. In a community-based study conducted by Brickman, Siedlecki et al. (2011), the researchers found that higher reserves are connected with better cognitive function performance such as memory, visual spatial ability and executive ability. Meanwhile, the same study suggested that people with higher reserves are better able to cope with cognitive diseases than those with lower reserves. Moreover, we identified interaction effects of education and age on executive function. A study based on 51 community-dwelling older adults found that education had a moderating effect on the influence of age on cognitive abilities including digital ability, reasoning ability, spatial orientation ability, verbal fluency and semantic comprehension, suggesting that higher educated participants have slower cognitive aging declines [32]. Our findings suggest that individuals with higher educational attainment have better cognition function in the cognitively normal stage. Additionally, the process of cognitive aging may be slower relative to their lower educated peers.

The results of the current study indicate that the highly educated elderly prefer to participate in knowledge-dependent activities such as reading, studying in a senior university, using the computer, and performing Chinese traditional martial arts, which were associated with lower risks of MCI [33]. Previous research demonstrated that reduced participation in leisure activities is an early marker of dementia that precedes declines on cognitive tests [34]. Verghese et al. reported that reading, playing board games, playing musical instruments, and dancing were associated with a reduced risk of dementia [35]. The lack of significant effects of playing musical instruments and dancing observed in our study is likely due to cultural differences. Cultural differences may influence this point because instrument playing and dancing is not popular among Chinese elders, whereas Chinese traditional martial arts, which serves as a type of conventional exercise in China, produces both physical and spiritual benefits. Educational experiences provide the foundation for continued intellectual stimulation across the life course, such as greater participation in various lifestyle activities. Further, participating in these knowledge-dependent activities provides a great opportunity for increasing cognitive ability. The education-cognition relations can be at least explained by participation in activities in later life.

With regard to the cognitive impairment stage, our post-hoc analysis showed that no cognitive tests exhibited a significant age×education interaction in MCI patients (Supplementary Table 3). Higher education levels may decrease the risk of MCI, and seem to confer protection leading to later cognitive impairment onset (p<0.001 for the mean age). MCI patients with higher education levels appeared to have poorer performance on the AVLT, BNT and TMT-B tests, although they did not have a significant age-related variance for any cognitive function. This finding strongly supports the viewpoint that education appears to have different impacts on CR in different cognitive stages [36]. Thus, in future studies, it will be necessary to consider the cognitive stage when exploring the protective effect of education. The GM results indicate that cognitively normal elders with low education suffer from more severe atrophy in the anterior brain regions (ORBsupmed.L and ACG.L) as age increases. Previous studies confirmed that the cognitively normal elderly with low levels of education suffer from more severe temporal and anterior region atrophy. For instance, Liu et al. found significantly greater thickness in the temporal pole, the transverse temporal gyrus, and the isthmus of the cingulate cortex among the elderly with higher levels of education [36]. Additionally, the anterior regions are considered to develop later and age earlier [37, 38]. These regions are more significantly affected by normal aging rather than AD, and the difference is significantly correlated with years of education [39]. Thus, obtaining more education experience could slow down the aging process in these regions.

The current study demonstrates that as the years of education increase, the WM integrity of the forceps major was reserved more and of the SLF.temporal part remained intact. The occipital lobe is vulnerable in the aging process similar to the prefrontal lobe [40]. Furthermore, a case study indicates that the forceps major may be related to the orientation ability [41]. Additionally, the results from structural equation modeling revealed interaction between age and education could affect cognition indirectly through SLF.temporal part integrity integrity. The SLF is a large bundle of association fibers in the WM of the cerebral hemispheres connecting the parietal, occipital and temporal lobes with the ipsilateral frontal cortices [42]. There is accumulating evidence that the SLF is correlated with core cognitive processes in the brain such as attention, language, emotions and memory. The SLF have extensive connections with cortical regions involved in language comprehension (Wernicke's area) and language production (Broca's area) [43]. Furthermore, patients with an SLF lesion or degeneration often show impairments in sentence processing and verbal working memory [44, 45]. To summarize, education may increase WM integrity among the healthy elderly, leading to increased brain reserves.

### Limitations

Our study has some limitations. Firstly, we only recruited Chinese elders who lived in the city and had a minimum of primary education. It is also important to investigate the effects of education in rural Chinese populations and to determine whether education influences the regional GM and WM differently among those with less than 6 years of schooling. Secondly, the elderly ranged in age from 48 to 87 years in our cognition sample, and from 60 to 81 years in the MRI sample. Further studies with a larger age span are needed to evaluate this interaction. Thirdly, data from prospective population-based autopsy studies of older individuals have suggested that the protective effect of education varied by brain pathological severities or cognitive stages [46]. The present study only focused on cognitively normal elderly, and did not include patients with cognitive impairments or AD. Furthermore, we did not rule out the role of occupation and income, which are the factor that is largely responsible for associations between education and later life cognitive functioning. Finally, our way of categorizing study participants into high/low education classes was only a proxy, it cannot reflect all aspects of latent and more abstract ‘education’.

## METHODS

### Participants

Participants in the present study were from the BABRI database, which is an ongoing longitudinal study that plans to collect cognitive behavioral, sociodemographics, lifestyle, potential influencing factor, and clinical data from Beijing’s elderly adults in five time-windows over 10 years, with more than 1600 eligible urban participants. The study was undertaken after obtaining the understanding and written consent of each subject, and the study conformed to the guidelines set out in the 2013 World Medical Association Declaration of Helsinki. The use of human subjects for this study has been approved by the institutional review board (IRB) at the Imaging Center for Brain Research at Beijing Normal University. Written informed consent was obtained from each participant.

A total of 1211 urban elderly living in Beijing, China were included in the BABRI database. Finally, 659 participants were enrolled in the present study (Supplementary Figure 1). These individuals included all right-handed native Han Chinese. Participants qualified for this study if they met the following criteria: 1) MMSE≥24; 2) no history of neurologic, psychiatric, or systemic illnesses known to influence cerebral function, including serious vascular diseases, head trauma, tumor, current depression, alcoholism, and epilepsy; 3) having completed 6 or more years of education; and 4) without a history of using psychoactive medications. People with suspected MCI or AD were excluded from the study. The diagnostic criteria for MCI was stated in Petersen [47]. The protocol was approved by the Institutional Review Board of the State Key Laboratory of Cognitive Neuroscience and Learning, Beijing Normal University. Written consent was obtained from each subject. Education level was assessed at baseline by recording the number of years of schooling. Two education levels were defined: the low education level (≤12 years) and the high education (>12 years) level.

### Neuropsychological testing

All participants underwent a battery of neuropsychological tests which was comprised of the following 5 cognition domains with the tests included in parentheses: 1) memory (Auditory Verbal Learning Test (AVLT), Rey-Osterrieth Complex Figure test (ROCF)(recall) and backward Digit Span); 2) attention (Trail Making Test (TMT) A, Symbol Digit Modalities Test (SDMT) and Stroop Color and Word Test (SCWT)-B); 3) visuo-spatial ability (ROCF (copy), Clock-Drawing Test (CDT)); 4) language (Category Verbal Fluency Test (CVFT), Boston Naming Test (BNT)); and 5)executive function (TMT-B and SCWT-C). The specific neuropsychological test procedures have been described previously [27].

### Analysis of genotyping

DNA was extracted from the subjects’ blood samples according to standard procedures for the subsequent characterization of the *APOE* genotype using PCR (Applied Biosystems, Foster City, CA). All participants were genotyped for two SNPs in the *APOE* gene (rs429358 and rs7412) using previously published methods [48].

### The measurement of leisure activity

Habitual leisure activity levels were measured using a personal information questionnaire, which is a subjective measure including 23 questions that ask participants to recall their leisure activity in the past one year. Leisure activities were defined as activities performed for enjoyment; these activities were independent of work and included reading; writing; participating in a senior citizens’ university; playing chess, poker, or mahjong; and handcrafts. The frequency of participation in each of the above activities was defined as frequent if the subject participated several times per week and rare if the subject participated less than once per week. Additionally, each leisure item was grouped according to intellectual, physical and social factors, and three aggregate scores were assigned to each participant for the subsequent analysis. In an attempt to create a valid data set, we excluded incomplete questionnaires.

### MRI data acquisition

Among the participants, 78 subjects (41 elderly with low education and 37 elderly with high education) received high-quality MRI scanning, which included a 3D T1-weighted MRI scan and a DTI scan. The MRI data were acquired using a SIEMENS TRIO 3T scanner in the Imaging Center for Brain Research at Beijing Normal University. Participants laid supine with their head fixed snugly by straps and foam pads to minimize head movement. T1-weighted, sagittal 3D magnetization prepared rapid gradient echo (MP-RAGE) sequences were acquired and covered the entire brain [176 sagittal slices, repetition time (TR)=1900 ms, echo time (TE)=3.44 ms, slice thickness=1 mm, flip angle=9°, inversion time=900 ms, field of view (FOV)=256×256 mm^2^, acquisition matrix=256×256]. For each DTI scan, images covering the whole brain were acquired by an echo-planar imaging sequence with the following scan parameters: TR=9500 ms, TE=92 ms, 30 diffusion-weighted directions with a b-value of 1000 s/mm^2^, and a single image with a b-value of 0 s/mm^2^, slice thickness=2 mm, no inter-slice gap, 70 axial slices, acquisition matrix =128×128, FOV=256×256 mm^2^, averages=3.

### Diffusion MRI data processing

DTI data were processed with the FDT toolbox in FSL (www.fmrib.ox.ac.uk/fsl). First, the DICOM files of all subjects were converted into NIfTI using the dcm2nii tool embedded in MRIcron. Second, the brain mask was estimated, and the resulting brain mask was used for the subsequent processing steps. Third, the non-brain spaces in the raw images were cut off leading to a reduced image size, reducing the memory cost and speeding up the processing in subsequent steps. Fourth, each image was coregistered to its corresponding b0 image using an affine transformation to correct the eddy-current induced distortions and simple head-motion artifacts. The diffusion gradient directions were adjusted accordingly. Fifth, a voxel-wise calculation of the tensor matrix and the diffusion tensor metrics were yielded for each subject, including the FA and mean diffusivity (MD).

To identify major white matter tracts, we used the digital WM atlas JHUICBM-DTI-81 (http://cmrm.med.jhmi.edu/), a probabilistic atlas generated by mapping the DTI data of 78 subjects to a template image. The atlas contains 20 main WM bundles and has three individual sets of sub-templates with different probability levels in the probability tractography map: 0%, 25% and 50%. In this study, we chose to use the 25% threshold subtemplate, which contains 20 major tracts. The JHU-WM atlas was overlaid on the WM skeleton of each subject in the CBM-DTI-81 space, such that each skeleton voxel could be categorized into one of the major tracts. Then, the FA and MD at the skeleton voxels within each tract were calculated.

### T1-weighted MRI data processing

All subjects’ individual structural images (3D T1-weighted anatomical images) were processed using theVBM8 toolbox of SPM (http://dbm.neuro.uni-jena.de/vbm/). The transformed T1-weighted images were then segmented and spatially normalized into GM, WM and cerebrospinal fluid in standard MNI space using a unified segmentation algorithm [49]. Modulation was further applied to the GM images to compensate for the effect of spatial normalization using linear and nonlinear methods.

ROIs were defined by the parcellation of the brain into the 90 regions of the automated anatomical labeling (AAL) template based on T1-weighted images. Each ROI volume for each subject was obtained by averaging the voxel values over the GM maps across all voxels within each AAL region.

### Statistical analysis

Independent two-sample t-tests were used to assess between-group differences in age and education. The chi-square test was used to compare proportional differences in the gender, *APOE* ε4 genotype, and frequency of leisure activities. We then assessed the effect of the age×education interaction on each cognitive test, and the aggregate leisure activity scores (intellectual, physical and social scores). Specifically, a GLM with “age”, “education” and “age×education” as predictor variables was used, wherein gender and *APOE*ε4 genotype were included as covariates.

We further investigated whether the expected education-related cognitive performance was mediated or moderated by leisure activity alterations in the large sample. To this aim, the mediation analyses were performed by the Hayes Process macro [50] (http://www.afhayes.com/). A mediator model typically consists of one independent variable, a mediator variable, and a dependent variable. This macro runs a series of ordinary least-squares regressions with the centered product term. In the present study, education constitutes the independent variable, cognitive measure was the dependent variable and aggregate leisure activity scores were the mediating variable. Thus, we would assume that the association between education and cognitive performance is mediated through the effect of education on leisure activity (i.e., an indirect effect). The significance threshold for the Sobel test was set at p<0.05. Structural equation modeling was employed to examine the association among education, age, cognition, gray matter volume and white matter integrity by Lisrel.

## CONCLUSION

In conclusion, our results demonstrate that the highly educated elderly have a higher level of wide cognitive function, higher frequencies of knowledge-related leisure activities, and smaller age-related anterior and temporal regional GM/WM integrity declines during aging. It is worthwhile to note that intellectual and social types of leisure activities mediate the association between education and multiple cognitive domains, including memory, language, attention and executive function. These novel findings suggest the important role of education in preventing aging and provide valuable insight into how high levels of education early in life facilitate the resistance of the neural system to cognitive aging.

## Supplementary Material

Supplementary Figures

Supplementary Tables
