# Supplementary material for: The positive impacts of early-life education on cognition, leisure activity, and brain structure in healthy aging
Source: Aging (Albany NY). 2019 Jul 17;11(14):4923–42. doi: 10.18632/aging.102088 (PMC6682517; doi:10.18632/aging.102088)
Supplement: Supplementary Figures [file aging-11-102088-s001.pdf]

SUPPLEMENTARY FIGURES

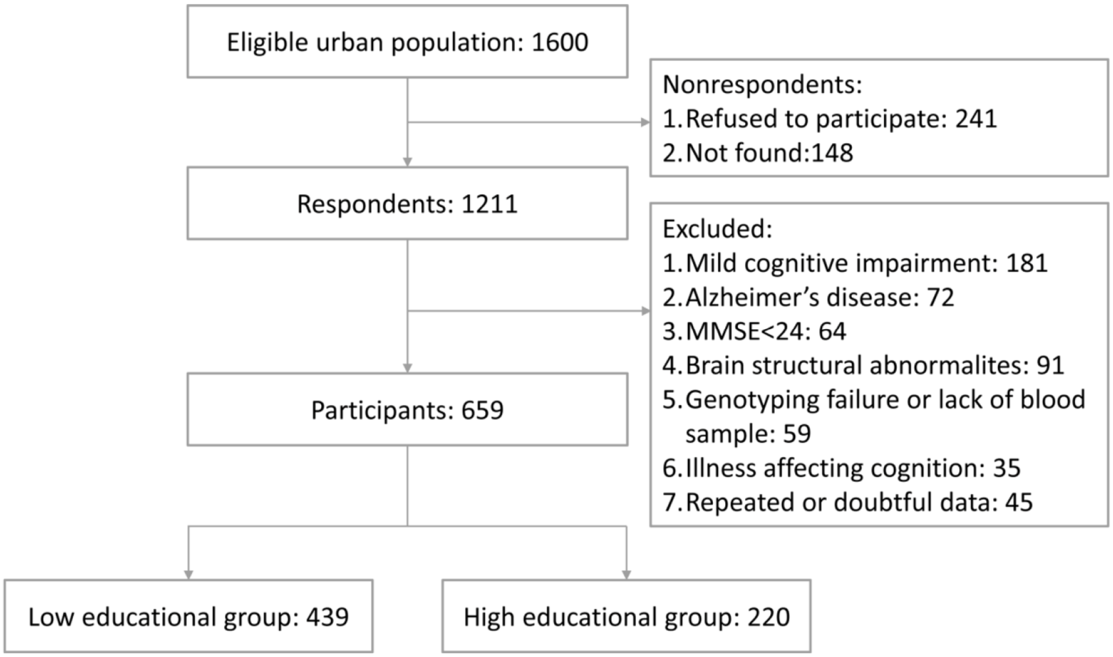

Supplementary Figure 1. A flow diagram of the study population.

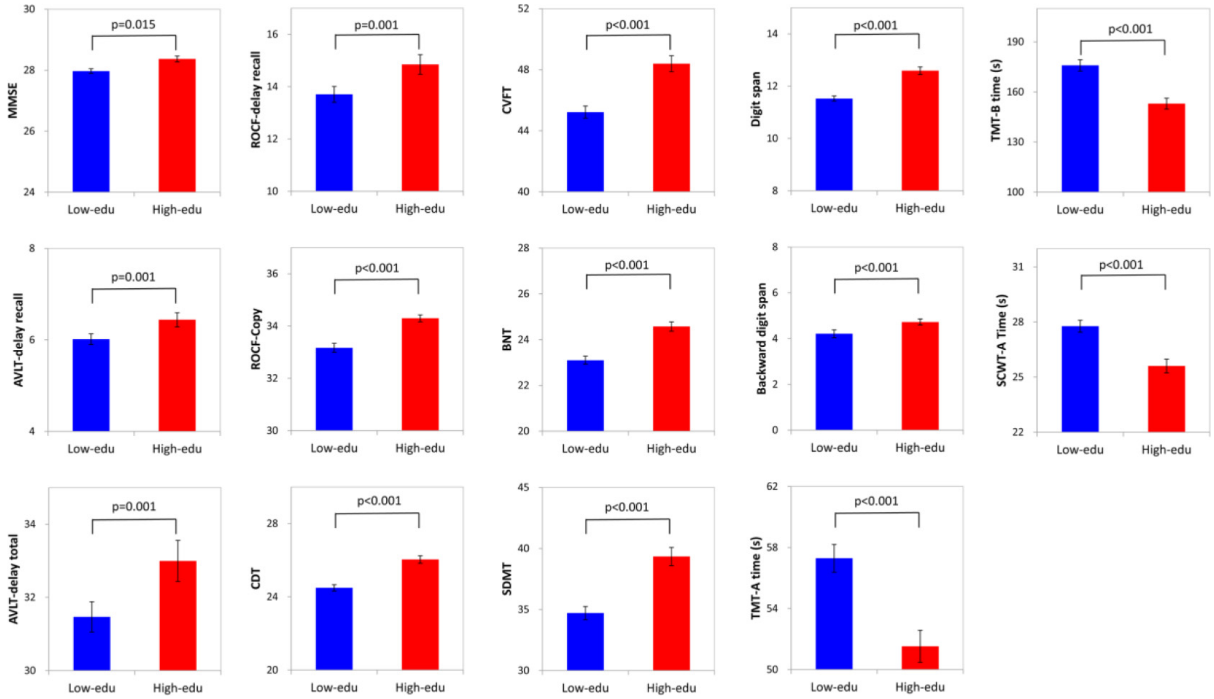

Supplementary Figure 2. The main effect of education on cognitive performance. Significant group differences of the cognitive tests between the low and high education groups. Error bars represent the standard error. MMSE=Mini-Mental Status Examination; AVLT=Auditory Verbal Learning Test; ROCF=Rey-Osterrieth Complex Figure test; CDT=Clock-Drawing Test; CVFT=Category Verbal Fluency Test; BNT=Boston Naming Test; SDMT=Symbol Digit Modalities Test; SCWT=Stroop Color and Word Test; TMT=Trail Making Test.

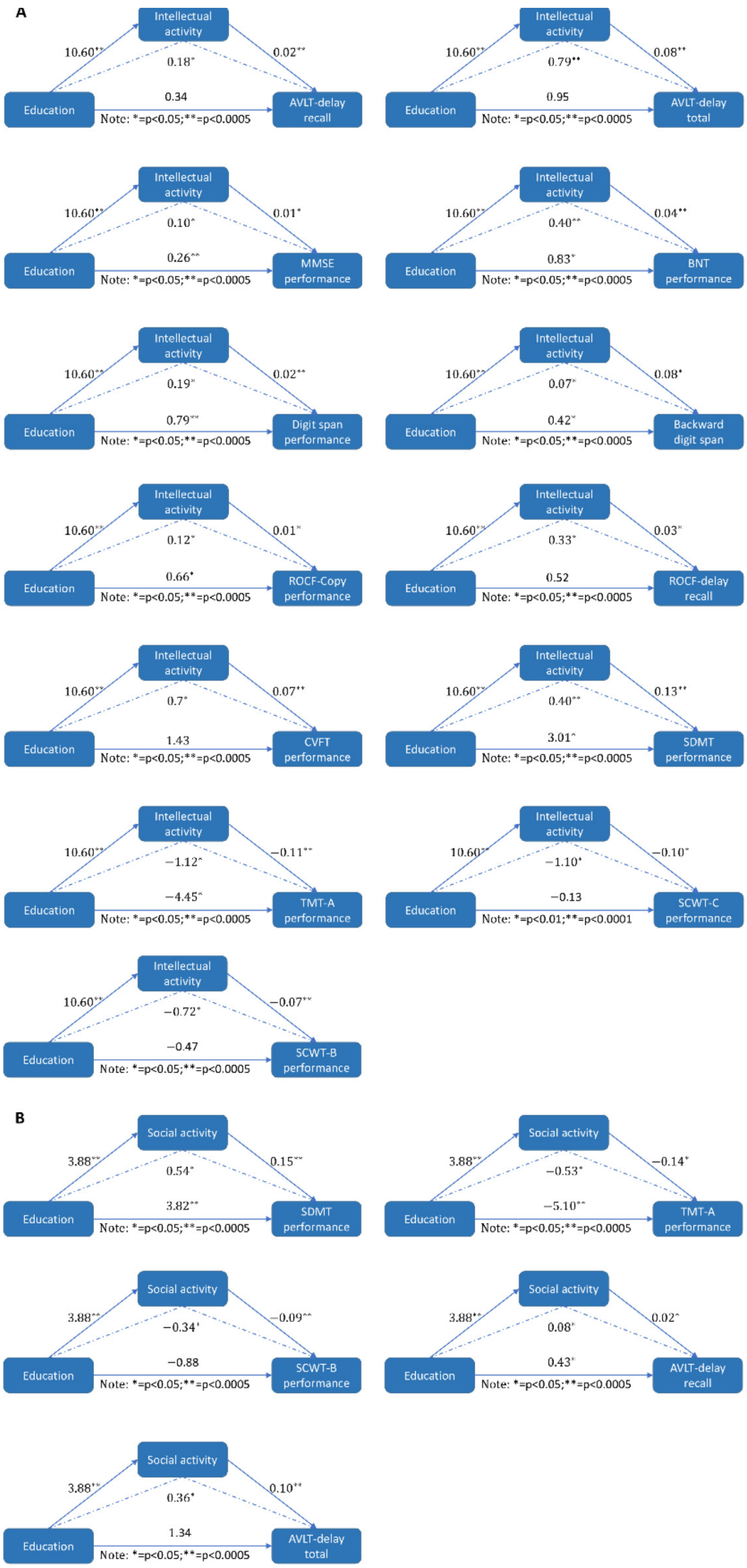

Supplementary Figure 3. The mediation model. (**A**) The mediation effect of intellectual activity education on multiple cognitive domains. (**B**) Mediating effect of social activity on the association between educational levels and memory, attention and executive function.

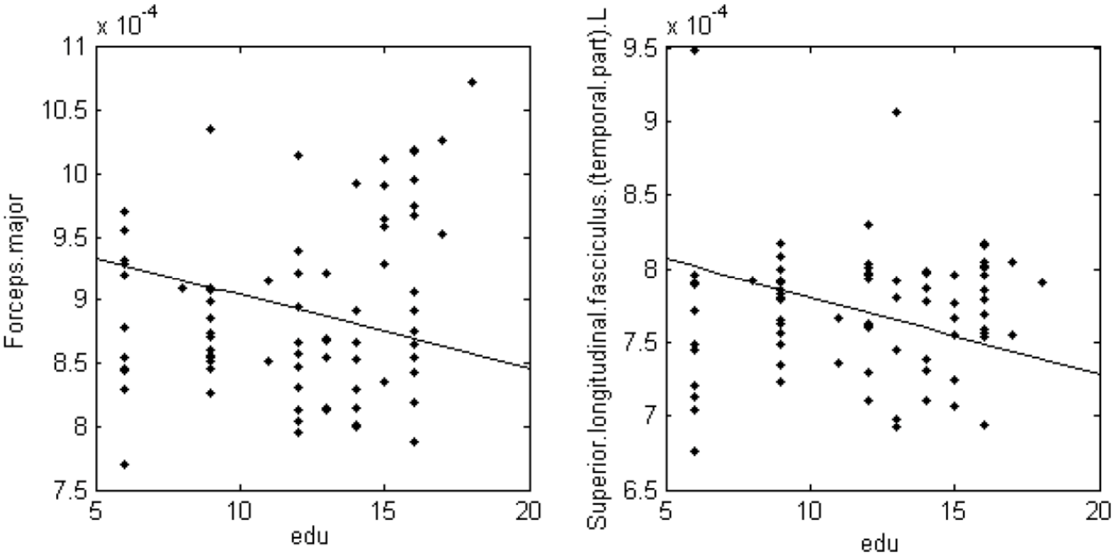

Supplementary Figure 4. Scatterplots showing the relationship between years of education and the MD values of the forceps major and the SLF.temporal part. MD=mean diffusivity; SLF=superior longitudinal fasciculus.
