# Supplementary material for: The positive impacts of early-life education on cognition, leisure activity, and brain structure in healthy aging
Source: Aging (Albany NY). 2019 Jul 17;11(14):4923–42. doi: 10.18632/aging.102088 (PMC6682517; doi:10.18632/aging.102088)
Supplement: Supplementary Tables [file aging-11-102088-s002.pdf]

SUPPLEMENTARY TABLES

Supplementary Table 1. Relationship between educational level and the leisure activity questionnaire.

leisure activity and frequency	Low Edu (n,%)	High Edu (n,%)	X^2	p
Reading				
Rare	45(12.8)	11(6.8)	4.024*	0.048
Frequent	307(87.2)	150(93.2)		
Writing				
Rare	321(91.2)	126(78.8)	15.36*	<0.0001
Frequent	31(8.8)	34(21.2)		
Participating in senior citizen university				
Rare	332(94.3)	140(87.0)	8.14*	0.008
Frequent	20(5.7)	21(13.0)		
Playing chess, poker, or mahjong				
Rare	269(76.2)	136(85.0)	5.125*	0.026
Frequent	84(23.8)	24(15.0)		
Doing manufacture by hand				
Rare	302(86.3)	151(95.0)	8.418*	0.003
Frequent	48(13.7)	8(5.0)		
Doing calligraphy, painting, or taking photos				
Rare	314(89.7)	138(85.7)	1.728	0.233
Frequent	36(10.3)	23(14.3)		
Playing a musical instrument, traditional Chinese opera or singing				
Rare	267(76.5)	113(71.5)	1.440	0.268
Frequent	82(23.5)	45(28.5)		
Watching TV or listening to radio				
Rare	12(3.4)	3(1.9)	0.948	0.409
Frequent	338(96.6)	158(98.1)		
Using a computer				
Rare	247(70.4)	78(48.4)	22.88*	<0.0001
Frequent	104(29.6)	83(51.6)		
Doing corssword puzzles, magic cube, or solitaire				
Rare	307(87.7)	128(81.0)	3.973	0.055
Frequent	43(12.3)	30(19.0)		
Doing aerobic exercise				
Rare	42(12.0)	16(9.9)	0.512	0.55
Frequent	307(88.0)	146(90.1)		
Doing muscular undurance sports				
Rare	317(90.8)	144(89.4)	0.245	0.63
Frequent	32(9.2)	17(10.6)		
Dancing or doing eurythmics				
Rare	220(62.7)	101(62.7)	<0.0001	1
Frequent	131(37.3)	60(37.3)		
Doing Chinese traditional martial arts				
Rare	298(84.2)	121(75.2)	5.944*	0.02
Frequent	56(15.8)	40(24.8)		
Climbing mountains, skiing, picking or fishing				
Rare	298(84.7)	138(86.3)	0.220	0.689

Frequent	54(15.3)	22(13.7)		
Travelling				
Rare	342(98.3)	155(98.1)	0.019	1
Frequent	6(1.7)	3(1.9)		
Rare	324(93.6)	148(91.4)	0.874	0.358
Frequent	22(6.4)	14(8.6)		
Planting				
Rare	155(44.0)	75(46.3)	0.230	0.635
Frequent	197(56.0)	87(53.7)		
Keeping pets				
Rare	214(61.5)	121(74.7)	8.542*	0.004
Frequent	134(38.5)	41(25.3)		
Visiting relatives and friends				
Rare	266(75.8)	124(76.5)	0.035	0.912
Frequent	85(24.2)	38(23.5)		
Attending a party				
Rare	316(92.1)	147(90.2)	0.537	0.496
Frequent	27(7.9)	16(9.8)		
Doing housework				
Rare	19(5.4)	8(4.9)	0.054	1
Frequent	333(94.6)	155(95.1)		
Babysitting				
Rare	251(71.9)	109(67.3)	1.142	0.299
Frequent	98(28.1)	53(32.7)		

Supplementary Table 2. Demographic and neuropsychological tests of MRI sample participants.

	Low Edu	High Edu	p(Edu)	p(Edu*Age)
Num	41	37	—	—
Age	66.63(6.12)	68.73(4.95)	0.102	—
Gender(M/F)	19/22	19/18	0.663	—
Education(year)	9.07(2.27)	15.00(1.31)	<0.0001	—
APOE ε4 (+/-)	14/27	11/26	0.681	—
MMSE	27.61(1.74)	28.05(1.51)	0.235	0.340
AVLT-delay recall	4.80(2.86)	5.43(2.74)	0.326	0.234
AVLT-delay total	27.80(9.71)	30.49(10.22)	0.238	0.484
ROCF-Copy	32.78(5.67)	34.43(1.68)	0.081	0.266
ROCF-delay recall	12.68(7.04)	13.46(5.90)	0.601	0.761
CDT	24.27(3.19)	26.82(3.17)	0.0009	0.031
CVFT	44.61(8.81)	49.23(8.03)	0.020	0.183
BNT	23.23(3.70)	24.26(3.31)	0.211	0.760
SDMT	31.38(9.81)	36.49(14.23)	0.073	0.535
Digit span	7.55(1.47)	7.36(1.08)	0.547	0.973
Backward digit span	4.38(1.35)	4.85(1.54)	0.167	0.784
TMT-A time (s)	57.22(17.84)	57.51(24.72)	0.952	0.032
TMT-B time (s)	193.66(79.06)	157.53(53.66)	0.002	0.375
SCWT-A Time (s)	28.51(7.68)	28.17(7.56)	0.846	0.550
SCWT-B Time (s)	39.10(7.24)	36.34(7.75)	0.056	0.967
SCWT-C Time (s)	76.59(16.99)	79.83(29.58)	0.974	0.009

Values are mean±standard deviation or Nos. of participants.
MMSE=Mini-Mental Status Examination; AVLT=Auditory Verbal Learning Test; ROCF=Rey-Osterrieth Complex Figure test; CDT=Clock-Drawing Test; CVFT=Category Verbal Fluency Test; BNT=Boston Naming Test; SDMT=Symbol Digit Modalities Test; SCWT=Stroop Color and Word Test; TMT=Trail Making Test.

Supplementary Table 3. Demographic and neuropsychological tests of MCI patients.

	Low Edu	High Edu	p(Edu)	p(Edu*Age)
Num	97	25	—	—
Age	61.84(6.28)	69.96(4.42)	<0.0001	—
Gender(M/F)	33/64	12/13	0.25	—
Education(year)	9.68(1.51)	15.88(1.39)	<0.0001	—
APOEε4 (+/-)	21/76	2/23	0.16	—
MMSE	26.53(1.99)	27.00(1.91)	0.08	0.84
AVLT-delay recall	3.08 (1.94)	4.38(2.96)	0.004	0.18
AVLT-delay total	20.88(7.19)	25.96(12.39)	0.009	0.19
ROCF-Copy	31.67(4.10)	32.88(3.03)	0.99	0.91
ROCF-delay recall	9.46(6.12)	12.52(7.68)	0.37	0.19
CDT	23.14(4.16)	24.26(4.00)	0.28	0.10
CVFT	39.28(7.79)	40.54(9.97)	0.88	0.25
BNT	21.21(3.74)	23.92(3.78)	0.05	0.59
SDMT	28.93(9.19)	28.65(10.16)	0.11	0.62
Digitspan	10.86(2.10)	11.13(1.96)	0.53	0.97
Backward digit span	3.88 (1.18)	4.17 (0.96)	0.28	0.95
TMT-A time (s)	73.01(29.24)	82.13(23.11)	0.88	0.61
TMT-B time (s)	225.79(73.73)	222.55(66.62)	0.04	0.82
SCWT-A Time (s)	30.97(8.36)	29.58(6.48)	0.14	0.74
SCWT-B Time (s)	44.08(12.34)	45.58(9.53)	0.12	0.50
SCWT-C Time (s)	89.56(28.06)	94.04(35.50)	0.06	0.74

Of the 181 patients with MCI, 59 patients were excluded due to genotype failure or lack of blood sample (n = 35), cerebrovascular lesions (n = 10), and doubtful data (n=14).
Values are mean±standard deviation or Nos. of participants.
MMSE=Mini-Mental Status Examination; AVLT=Auditory Verbal Learning Test; ROCF=Rey-Osterrieth Complex Figure test; CDT=Clock-Drawing Test; CVFT=Category Verbal Fluency Test; BNT=Boston Naming Test; SDMT=Symbol Digit Modalities Test; SCWT=Stroop Color and Word Test; TMT=Trail Making Test.
